# Molecular Mechanisms of Dysregulated LH and FSH Secretion in Human Reproductive Failure

**DOI:** 10.3390/biomedicines14040789

**Published:** 2026-03-31

**Authors:** Athanasios Zikopoulos, Efthalia Moustakli, Anastasios Potiris, Vasilis Sebastian Paraschos, Periklis Katopodis, Pavlos Machairoudias, Panagiotis Antsaklis, Nikolaos Kathopoulis, Ismini Anagnostaki, Sofoklis Stavros

**Affiliations:** 1 Department of Reproductive Medicine and Surgery, University College London Hospitals NHS Foundation Trust, 235 Euston Road, London NW1 2BU, UK; 2 Department of Nursing, School of Health Sciences, University of Ioannina, 4th Kilometer National Highway Str. Ioannina-Athens, 45500 Ioannina, Greece; 3 Third Department of Obstetrics and Gynecology, University General Hospital “ATTIKON”, Medical School, National and Kapodistrian University of Athens, 12462 Athens, Greece; apotiris@med.uoa.gr (A.P.); sfstavrou@med.uoa.gr (S.S.); 4 Department of Obstetrics and Gynecology, Corewell Health Hospital, 4700 Schaefer Road Suite 310, Dearborn, MI 48126, USA; vasilispar10@gmail.com; 5 Laboratory of Medical Genetics in Clinical Practice, Faculty of Medicine, School of Health Sciences, University of Ioannina, 45110 Ioannina, Greece; 6 Oxford University Hospitals NHS Foundation Trust, Oxford OX3 9DU, UK; 7 First Department of Obstetrics and Gynecology, Alexandra Hospital, Medical School, National and Kapodistrian University of Athens, 11528 Athens, Greece; pantsak@med.uoa.gr (P.A.); nickatho@med.uoa.gr (N.K.); 8 Medical School, National and Kapodistrian University of Athens, 11528 Athens, Greece; isanagnostaki3@gmail.com

**Keywords:** GnRH pulsatility, KNDy neurons, GNRHR signaling, LHB and FSHB transcription, activin–inhibin axis, SMAD pathway, polycystic ovary syndrome, hypogonadotropic hypogonadism

## Abstract

Several reproductive issues in both men and women are caused by changes in the pulsatile secretion of luteinizing hormone (LH) and follicle-stimulating hormone (FSH). For males to sustain spermatogenesis and Leydig cell function, and for females to ensure orderly folliculogenesis, ovulation, and ovarian steroidogenesis, precise coordination of LH and FSH secretion is necessary. Pituitary responsiveness, the frequency or amplitude of gonadotropin-releasing hormone pulses, or the dysregulation of feedback signals mediated by sex steroids and inhibins all disrupt the balance between LH and FSH secretion. Oligozoospermia, luteal-phase abnormalities, anovulation, or complete spermatogenic failure are possible clinical signs of these alterations. In addition to functional neuroendocrine disturbances, emerging genetic and epigenetic evidence, including pathogenic variants in genes such as gonadotropin-releasing hormone receptor, kisspeptin, kisspeptin receptor, luteinizing hormone beta subunit, follicle-stimulating hormone beta subunit, follicle-stimulating hormone receptor, and luteinizing hormone/choriogonadotropin receptor, has highlighted the role of inherited and acquired molecular defects in disrupting gonadotropin regulation. This narrative review synthesizes contemporary mechanistic, clinical, translational, and genetic evidence elucidating how dysregulated secretion of LH and FSH contributes to reproductive dysfunction. The molecular processes that regulate gonadotropin synthesis and release, as well as neuroendocrine regulation, gene-level determinants of hypothalamic–pituitary–gonadal (HPG) axis dysfunction, and the clinical phenotypes that result from their disruption, are all given special attention. We conclude with a discussion of new treatment strategies that target local intragonadal regulators to enhance gametogenic capacity, modulate gonadotropin signaling, or restore physiological gonadotropin-releasing hormone (GnRH) pulsatility, with consideration of how genetic insights may inform personalized therapeutic approaches.

## 1. Introduction

The hypothalamic–pituitary–gonadal (HPG) axis is a highly integrated neuroendocrine network that converts central neuronal inputs into gonadal steroidogenesis and gametogenesis. Its perfect coordination is what determines reproductive competence in both sexes [1]. The pulsatile release of GnRH from certain hypothalamic neurons is essential to this control. In pituitary gonadotrophs, the frequency and amplitude of gonadotropin-releasing hormone (GnRH) pulses function as a biological code that variably regulates the transcription of the follicle-stimulating hormone beta (*FSHB*) and luteinizing hormone beta (*LHB*) subunits [2,3]. Pulsatile GnRH signaling coordinates selective gonadotropin synthesis and release through activation of G protein–coupled GnRH receptors. Reproductive physiology can be significantly impacted by even little changes in this temporal signaling pattern, which can disrupt the luteinizing hormone/follicle-stimulating hormone (LH/FSH) balance [3].

In addition to hypothalamic regulation, steroid and peptide feedback mechanisms dynamically modulate the function of pituitary gonadotropic cells. GnRH receptor expression and intracellular signaling are modulated by estradiol, progesterone, and testosterone, while *FSHB* subunit transcription is controlled by the activin–inhibin–follistatin system through SMAD-dependent pathways [4]. These regulatory loops ensure precise coupling between gonadotropin secretion and gonadal function, enabling processes such as follicle selection, ovulatory capacity, corpus luteum development, intratesticular testosterone production, and the progression of spermatogenesis [5].

A wide range of reproductive disorders result from the dysfunction of these tightly and intricately regulated molecular and neuroendocrine processes. The increased pulsatility of GnRH, which is often observed in women with polycystic ovary syndrome, favors the production of LH at the expense of FSH, thus preventing the selection of the dominant follicle and promoting persistent anovulation [6,7]. Conversely, the reduced pulsatility of GnRH leads to suppression of the secretion of both FSH and LH, with subsequent hypoestrogenism and functional ovarian inactivity, as characteristically observed in functional hypothalamic amenorrhea [8,9,10].

In addition to functional neuroendocrine disturbances, genetic abnormalities affecting key components of the HPG axis have been identified as major contributors to reproductive disease [11]. Beyond their effects on GnRH pulsatility and gonadotropin synthesis, monogenic and polygenic variants may modify receptor signaling dynamics and transcriptional regulation, ultimately resulting in heterogeneous reproductive phenotypes [12,13,14].

In men, inadequate gonadotropic signaling disrupts the steroidogenic pathways of Leydig cells and the supportive function of Sertoli cells during germ cell maturation [15,16]. At the same time, increasing data indicate that these pathological conditions are influenced by metabolic signals, dysregulation of the kisspeptin/neurokinin B/dynorphin (KNDy) neuronal network, as well as epigenetic mechanisms that modify gonadotropin gene expression [17].

This narrative review synthesizes recent advances in cellular, molecular, genetic and translational research, aiming to elucidate the mechanisms that lead to pathological FSH and LH signaling. It also highlights how disruptions in these interconnected regulatory pathways result in reproductive dysfunction, examining alterations in GnRH pulsatility, pituitary signal transduction, transcriptional regulation of gonadotropin subunits, and gonadal feedback mechanisms. Unlike previous reviews, this work integrates intracellular signaling dynamics with genetic determinants of gonadotropin dysregulation, emphasizing how GnRH pulse decoding and receptor-mediated signaling are translated into distinct reproductive phenotypes and variable therapeutic responses. Importantly, we further conceptualize these processes within an integrative framework in which GnRH pulsatility, pituitary signal decoding, and gonadal feedback act as interconnected regulatory layers rather than isolated mechanisms. Within this model, genetic and epigenetic modifiers operate across multiple levels of the HPG axis, contributing to phenotypic variability and differential clinical expression. Finally, emerging therapeutic strategies that selectively target critical nodes of the HPG axis are discussed.

## 2. Literature Search Strategy

This narrative review summarizes the molecular and neuroendocrine mechanisms underlying dysregulation of LH and FSH secretion and their contribution to human reproductive failure. To identify relevant literature, structured searches were performed in the PubMed/MEDLINE, Scopus, and Web of Science databases, using keyword combinations such as “GnRH pulsatility,” “gonadotropin signaling,” “LHβ transcription,” “FSHB gene regulation,” “GnRH receptor signaling,” “activin–inhibin–follistatin system,” “KNDy neurons,” “polycystic ovary syndrome,” “functional hypothalamic amenorrhea,” “hypogonadotropic hypogonadism,” “folliculogenesis,” and “spermatogenesis,” as well as “genetic mutations,” “gene variants,” “*GNRHR* mutations,” “*KISS1*/*KISS1R* variants,” “*FSHR* polymorphisms,” and “*LHCGR* mutations.”

The review focused on studies that shed light on intracellular signaling pathways, transcriptional regulation of gonadotropin subunits, feedback mechanisms within the HPG axis, gene-level determinants of gonadotropin dysregulation, and their translational associations with distinct reproductive phenotypes. Priority was given to original, peer-reviewed research studies, as well as high-quality reviews, published primarily between January 2000 and March 2025. To provide adequate historical and mechanistic context, older studies were also incorporated where necessary. Particular emphasis was placed on recent high-impact studies (2020–2025) to ensure that the review reflects current advances in GnRH pulsatility, gonadotropin signaling, and genetic regulation of the HPG axis.

The references of the selected articles were manually reviewed to identify additional relevant publications. The retrieved material was organized thematically based on clinical features, neuroendocrine regulation, genetic determinants, and underlying molecular mechanisms. This review was prepared without generating or analyzing new experimental data.

## 3. Physiology of LH and FSH Secretion

**Figure 1:**
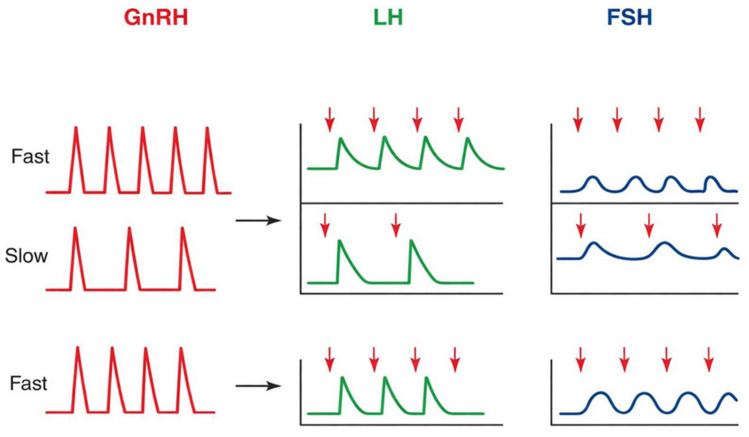
GnRH pulse frequency–dependent regulation of gonadotropin secretion. The X-axis represents time, and the Y-axis indicates relative hormone secretion levels. High-frequency GnRH pulses preferentially stimulate LH secretion, whereas low-frequency pulses favor FSH production. Abbreviations: GnRH, gonadotropin-releasing hormone; LH, luteinizing hormone; FSH, follicle-stimulating hormone [21].

In response to pulsatile stimulation by GnRH, the gonadotropic cells of the pituitary gland secrete LH and FSH. Variations in GnRH pulse frequency differentially regulate LH and FSH synthesis, with higher frequencies associated with LH predominance and lower frequencies with FSH predominance (Figure 1). This differential regulation is mediated by frequency-dependent activation of intracellular signaling pathways and transcription factors, such as *EGR1*, *NR5A1*, and *FOXL2*, which selectively modulate *LHB* and *FSHB* gene expression. Through this mechanism, the reproductive system dynamically adjusts its hormonal output to meet different physiological demands [3,5,18]. In parallel, the circulating sex steroids, inhibin, activin, and locally produced follistatin regulate the sensitivity and functional response of the gonadotropic cells [19,20]. Taken together, these regulatory inputs constitute an integrated feedback network that synchronizes gonadotropin secretion with the functional activity of the gonads.

**Figure 2:**
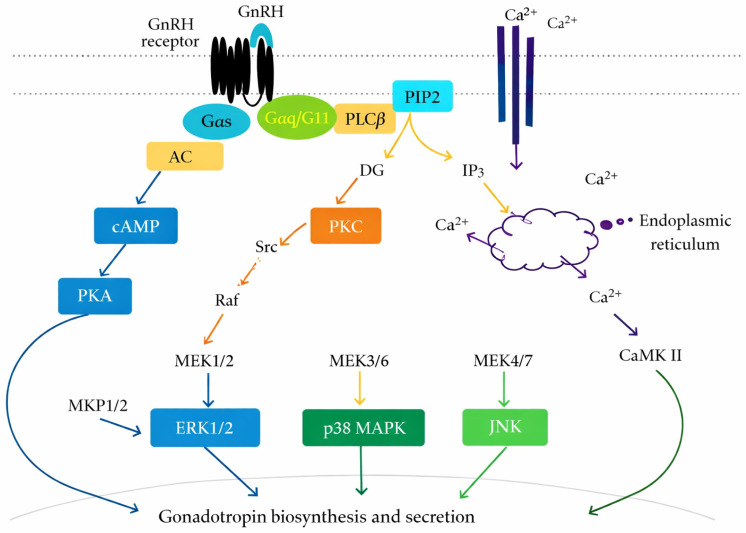
GnRH receptor-mediated intracellular signaling in pituitary gonadotrophs. GnRH binding activates G protein–coupled pathways, including cAMP/PKA, PLC/IP3/DAG, calcium signaling, and MAPK cascades (ERK, p38, JNK), leading to gonadotropin synthesis and secretion. Abbreviations: GnRH, gonadotropin-releasing hormone; cAMP, cyclic adenosine monophosphate; PKA, protein kinase A; PLC, phospholipase C; IP3, inositol trisphosphate; DG, diacylglycerol; MAPK, mitogen-activated protein kinase; ERK, extracellular signal-regulated kinase; JNK, c-Jun N-terminal kinase; p38, p38 mitogen-activated protein kinase [30].

The recruitment of ovarian follicles and the proliferation of granulosa cells in women are driven by FSH, whereas LH stimulates testosterone production in theca cells and starts ovulation during the preovulatory LH surge [22,23,24]. In men, FSH maintains Sertoli cell maturation and metabolic activity, which are essential for germ cell development, whereas LH stimulates testosterone production in Leydig cells. Therefore, balanced testosterone production and spermatogenesis in males, as well as regular folliculogenesis and ovulation in women, depend on the coordinated action of LH and FSH [25,26]. GnRH receptor activation initiates multiple intracellular signaling cascades, including PLC/PKC, calcium mobilization, and MAPK pathways, which collectively regulate gonadotropin gene transcription (Figure 2).

### 3.1. Central Regulation of GnRH Pulsatility

LH and FSH secretion are dependent on the pulsatile release of GnRH from specific hypothalamic neurons, which are mostly found in the mediobasal hypothalamus and preoptic region. A frequency-encoded biological signal that variably controls pituitary gonadotropin production is shown by this pulsatile pattern. LHβ transcription is preferentially stimulated by fast GnRH pulse frequencies, while FSHβ expression is favored by slower pulse frequencies. Thus, the hypothalamus’s ability to produce and regulate these rhythmic oscillations is essential for preserving reproductive competence [3].

KNDy neurons, a subset of arcuate nucleus neurons that co-express kisspeptin (encoded by *KISS1*), neurokinin B (*TAC3*), and dynorphin (*PDYN*), are critical regulators of GnRH pulse generation. These neurons combine excitatory and inhibitory transmission to produce a linked oscillating network [27]. While dynorphin, which acts through κ-opioid receptors, produces an inhibitory tone that ends each pulse, neurokinin B acts through neurokinin 3 receptors (NK3R) to increase coordinated neuronal activity. The main stimulatory output of this network is kisspeptin, which activates phospholipase C and mobilizes intracellular calcium to cause GnRH release via its Gq/11-coupled receptor KISS1R on GnRH neurons. During the reproductive cycle, circulating estradiol can dynamically alter pulse frequency through steroid feedback regulation mediated by estrogen receptor-α (ERα), which is expressed in KNDy neurons [28,29].

Environmental and physiological cues further control this pulse generator. Peripheral signals, such as leptin, insulin, and ghrelin, indirectly affect KNDy neuronal activity through intermediary hypothalamic circuits, which include proopiomelanocortin (POMC) and neuropeptide Y/agouti-related peptide (NPY/AgRP) neurons. GnRH pulsatility can be inhibited by stress-related neuropeptides and glucocorticoids that alter the excitability of the KNDy network [31,32]. Furthermore, recent studies have linked long-term changes in reproductive function during chronic stress or energy deprivation to epigenetic processes, including DNA methylation and histone modifications at the KISS1 and GNRH1 loci.

In addition to DNA methylation, histone modifications such as acetylation and methylation of regulatory regions influence chromatin accessibility at key neuroendocrine genes, thereby modulating transcriptional activity of *KISS1* and *GNRH1*. Emerging evidence also highlights the role of non-coding RNAs, including microRNAs, in post-transcriptional regulation of GnRH neuronal function and upstream signaling pathways. These epigenetic mechanisms provide a dynamic interface through which environmental factors, including nutritional status and chronic stress, can exert sustained effects on HPG axis activity [33].

Despite these advances, important uncertainties remain regarding the central regulation of GnRH pulsatility. Much of the current mechanistic model of pulse generation is derived from animal studies, particularly in rodents and sheep, whereas direct functional assessment in humans remains limited. In addition, although KNDy neurons are widely recognized as core components of the GnRH pulse generator, the relative contribution of upstream metabolic, stress-related, and steroid-dependent inputs may vary across physiological and pathological states. The extent to which epigenetic alterations act as primary drivers of GnRH dysregulation, rather than secondary adaptations to environmental or endocrine stress, also remains incompletely understood [34]. These limitations underscore the need for integrative human studies to better define how central pulse regulation is altered in reproductive disorders.

### 3.2. Pituitary Gonadotroph Function and Signal Transduction

The primary biological targets of GnRH are pituitary gonadotrophs, which convert pulsatile hypothalamic impulses into differential production and secretion of FSH and LH. GnRH attaches to its G protein–coupled receptor (GNRHR), which is primarily connected to the Gq/11 pathway. Phospholipase C-β (PLCβ) is stimulated by receptor activation, which causes phosphatidylinositol 4,5-bisphosphate (PIP2) to hydrolyze into inositol trisphosphate (IP3) and diacylglycerol (DAG). While DAG activates PKC, IP3 initiates intracellular calcium mobilization, which in turn starts downstream kinase cascades such as ERK1/2, JNK, and p38 MAPK [18,35,36].

The transcription of the genes encoding the gonadotropin component is controlled by these signaling pathways. While the β-subunits encoded by LHB and FSHB define their biological specificity, LH and FSH share a common α-subunit (CGA). By differentially engaging kinase pathways and transcription factors, GnRH pulse frequency selectively regulates gene transcription [37,38]. While prolonged signaling conditions promote FSHB expression, ERK-dependent activation of early growth response protein 1 (EGR1) and activator protein-1 (AP-1) components selectively increases LHB transcription [37].

The system of activin, inhibitin, and follistatin refines the production of FSH. Activin promotes the phosphorylation of type I receptors and the activation of SMAD2/3 by binding to type II activin receptors (ActRIIA/IIB). Translocating to the nucleus, the SMAD2/3–SMAD4 complex works with transcription factors, including FOXL2, to increase the activity of the FSHB promoter [4,39]. Follistatin binds activin extracellularly and reduces its bioavailability, whereas inhibin inhibits activin signaling through interactions with the co-receptor betaglycan. Acute GnRH stimulation is not necessary for these pathways to selectively modulate FSH production [40].

The response of gonadotrophs to GnRH is dynamically controlled. Prolonged GnRH stimulation promotes receptor internalization, β-arrestin recruitment, and receptor phosphorylation, resulting in receptor desensitization and diminished downstream signaling [41]. On the other hand, physiological pulsatility maintains prolonged transcriptional competence and the integrity of receptor signaling. Intracellular signal integration at the level of kinase activation and gene transcription represents a critical regulatory node within the HPG axis. Disruption of these processes can disturb LH and FSH homeostasis and impair reproductive function [42,43].

Despite the detailed characterization of these intracellular signaling pathways, their relative contribution to LH and FSH regulation in vivo remains context-dependent and incompletely defined. Much of the current understanding is based on in vitro systems or animal models, which may not fully recapitulate the complexity of human pituitary physiology [44]. In particular, the extent to which specific kinase pathways such as ERK versus SMAD signaling dominate under different GnRH pulse frequencies and endocrine environments remains an area of ongoing investigation. Furthermore, the integration of these signaling networks with genetic variability and epigenetic regulation is not yet fully understood. These limitations highlight the need for more physiologically relevant models and integrative approaches to better define how pituitary signal transduction contributes to gonadotropin imbalance in human reproductive disorders. For clarity, emphasis is placed on key signaling pathways with established biological and clinical relevance [30,43]. Table 1 summarizes the key molecular determinants distinguishing LH and FSH synthesis and regulation.

**Table 1:** Molecular factors that influence the regulation of LH and FSH differently. Pituitary gonadotrophs’ LHB and FSHB expression is regulated by transcription factors, intracellular signaling pathways, GnRH pulse decoding, and local modulators.

Feature	LH Regulation	FSH Regulation
GnRH pulse sensitivity [45]	High-frequency pulses	Low-frequency pulses
Key signaling pathway [46,47]	ERK1/2, PKC	SMAD2/3
Main transcription factors [48]	EGR1, AP-1	SMAD4, FOXL2
Gene target [47]	LHB	FSHB
Peptide regulation [37]	Minimal	Activin/Inhibin/Follistatin
Secretion pattern [49]	Pulsatile bursts	Gradual fluctuations
Associated genetic variants [50,51,52,53]	LHB, GNRHR, ERK pathway genes	FSHB, FSHR, SMAD pathway genes

### 3.3. Feedback Regulation by Sex Steroids and Intragonadal Peptides

Steroid and peptide feedback signals from the gonads, which act at the hypothalamus and pituitary levels, dynamically regulate the secretion of LH and FSH [53]. The primary mechanism by which ligand-activated nuclear receptors, namely estrogen receptors α and β (ERα/ERβ), progesterone receptors (PR-A and PR-B), and the androgen receptor (AR), are responsible for the actions of sex steroids, such as estradiol, progesterone, and testosterone. By binding to hormone response elements found in target gene promoters, these receptors act as transcription factors that alter the expression of genes related to gonadotropin subunit transcription, GnRH synthesis, and GnRH receptor expression [54,55,56].

Estradiol exerts context-dependent feedback effects on the HPG axis. Estradiol inhibits GnRH pulse frequency in hypothalamic KNDy neurons through ERα at low to moderate doses, which lowers LH secretion [57]. On the other hand, persistently elevated levels of estradiol in the late follicular phase set off positive feedback systems that improve the firing of GnRH neurons and encourage the preovulatory LH surge. By controlling the expression of the GNRHR, LHB, and FSHB genes and interacting with intracellular kinase pathways that affect transcription factor activity, estradiol affects gonadotroph sensitivity at the pituitary level [58]. By lowering the GnRH pulse frequency, progesterone aids in feedback regulation. This is partially due to changes in opioid peptide production and KNDy neuronal activity. In males, testosterone maintains intratesticular androgen homeostasis by exerting AR–mediated negative feedback on LH secretion at the hypothalamic and pituitary levels [5].

Intragonadal peptides offer specific control of FSH synthesis in addition to steroid-mediated regulation. By opposing activin signaling at the pituitary, inhibin, produced by granulosa cells in females and Sertoli cells in males, suppresses the synthesis of FSH. By interacting with activin type II receptors and the co-receptor betaglycan, this inhibitor stops SMAD2/3 from being phosphorylated and the FSHB promoter from being activated [4,59]. Activin enhances FSH synthesis by promoting SMAD2/3–SMAD4–mediated transcription, frequently in concert with transcription factors such as FOXL2. Follistatin enhances this mechanism by binding to activin extracellularly and decreasing its bioavailability. The selective control of FSH production and gonadal steroid status is integrated through cross-talk between SMAD signaling and steroid receptor pathways [47].

Throughout the reproductive life cycle, these peptide-mediated and genetic feedback systems cooperate to maintain the LH/FSH equilibrium. Anovulation, luteal dysfunction, impaired spermatogenesis, and other endocrine abnormalities can arise from disruptions in steroid receptor signaling, activin–inhibin balance, or SMAD-dependent transcriptional regulation, thereby compromising gonadotropin homeostasis [41,60].

### 3.4. Physiological Roles of LH and FSH in Females

Folliculogenesis, ovulation, and luteal function in the female reproductive system are regulated by the coordinated actions of LH and FSH via tightly controlled receptor-mediated signaling pathways. The principal effects of FSH on granulosa cell function are mediated via the cAMP–protein kinase A (PKA) pathway, which is activated by the FSHR, a G protein–coupled receptor. This signaling cascade increases the expression of aromatase (*CYP19A1*), enhances the manufacture of estradiol, and encourages the growth of granulosa cells. Furthermore, the LHCGR, which prepares the dominant follicle for LH responsiveness in the late follicular phase, is produced by maturing granulosa cells in response to FSH [26,61,62].

*LHCGR* is expressed by theca cells and subsequently by preovulatory granulosa cells, to which LH interacts. LH stimulates androgen production in theca cells by activating the steroidogenic acute regulatory protein (StAR) and key steroidogenic enzymes, including *CYP11A1* and *CYP17A1*, via the cAMP/PKA signaling pathway. These androgens serve as substrates for aromatization in granulosa cells. The dominant follicle is selected for and maintained in part by elevated levels of estradiol. The mid-cycle LH surge is caused by positive feedback processes that are activated after a critical threshold is reached [63,64,65,66].

The LH surge initiates a complex ovulatory cascade characterized by increased expression of prostaglandin-endoperoxide synthase 2 (*PTGS2*/COX-2), upregulation of epidermal growth factor–like ligands, including amphiregulin and epiregulin, and rapid activation of ERK1/2 signaling. Through modifications of cyclic AMP and maturation-promoting factor activity, these mechanisms promote follicular rupture, cumulus expansion, and the resumption of oocyte meiosis. The production of progesterone, which is necessary for endometrial receptivity, is then increased by LH’s promotion of the luteinization of theca and granulosa cells [67,68].

Changes in steroidogenic enzyme activity, interruption of FSHR or LHCGR signaling, or changed LH/FSH ratios can all affect follicular growth and ovulatory competence. Low aromatase activity, excess LH relative to FSH, or defective LH surge signaling can cause anovulation, luteal insufficiency, and decreased fertility [69].

### 3.5. Physiological Roles of LH and FSH in Males

LH and FSH use receptor-mediated signaling in Leydig and Sertoli cells to regulate androgen synthesis and spermatogenesis in the male reproductive system. LH triggers a Gs–cAMP–PKA cascade that promotes steroidogenesis by binding to the LHCGR on Leydig cells. The StAR and important mitochondrial and microsomal enzymes involved in testosterone production are expressed more frequently as a result of this pathway. Spermiogenesis, meiotic progression, and maintenance of the spermatogenic epithelium depend on high intratesticular testosterone concentrations [70,71].

FSH predominantly engages cAMP/PKA signaling and downstream phosphorylation of cAMP response element–binding protein (CREB) in Sertoli cells through the FSHR. The blood–testis barrier, metabolic cooperation, and germ cell survival are all supported by this transcriptional pathway. Moreover, FSH promotes the synthesis of androgen-binding protein and inhibin B, the latter of which is a crucial modulator of pituitary FSH release [72,73].

In Sertoli cells, testosterone mediates its actions via AR–dependent signaling pathways. Ligand-activated AR controls genes essential for spermatocyte development and the anatomical arrangement of the seminiferous epithelium [74]. Male fertility is largely dependent on the functional interdependence of pathways regulated by FSH and LH: FSH maximizes Sertoli cell responsiveness to androgens, while LH-driven testosterone synthesis improves AR signaling. Impaired LHCGR, FSHR, or AR signaling, as well as disrupted steroidogenic coordination, can compromise spermatogenesis, leading to oligozoospermia or spermatogenic arrest. Maintaining male reproductive capability thus requires precise molecular coordination between the FSH and LH pathways [25,75]. Table 2 summarizes the primary cellular targets and signaling pathways of FSH and LH in the gonads.

**Table 2:** Cellular targets and functional roles of LH and FSH in the gonads. Summary of the primary functional outcomes of gonadotropin signaling in ovarian and testicular tissues, including target cells, receptors, and intracellular signaling cascades.

Hormone	Sex	Target Cell	Receptor	Key Pathway	Main Effect	Clinical Relevance
LH [76,77]	Female	Theca cells	LHCGR	cAMP/PKA	Androgen synthesis	Ovulation and follicular development
LH [71,78]	Male	Leydig cells	LHCGR	cAMP/PKA	Testosterone production	Spermatogenesis support
FSH [79,80]	Female	Granulosa cells	FSHR	cAMP/PKA	Aromatase activity, estradiol synthesis	Folliculogenesis
FSH [75,81]	Male	Sertoli cells	FSHR	cAMP/CREB	Inhibin B production, germ cell support	Spermatogenesis

### 3.6. Temporal Dynamics and Interdependence of LH and FSH Secretion

Pituitary gonadotrophs’ capacity to translate pulsatile GnRH signals into distinct transcriptional outputs is reflected in the temporal pattern of LH and FSH production, which is a basic tenet of reproductive endocrinology [3]. LH and FSH show different sensitivity to pulse frequency and amplitude, despite the fact that both hormones are released in response to separate GnRH pulses. LHβ transcription is enhanced by rapid GnRH pulse frequencies, which preferentially activate ERK1/2-dependent signaling and stimulate transcription factors including EGR1 and AP-1. Slower pulse frequencies, on the other hand, promote longer signaling contexts that promote the production of FSHβ, in part due to enhanced activity of the activin–SMAD2/3–SMAD4 pathway. The selective control of gonadotropin component gene expression at the promoter level is made possible by this frequency decoding process [37,82].

Beyond rapid kinase activation, temporal dynamics also shape transcriptional kinetics and chromatin accessibility. Pulse-dependent gene responsiveness is influenced by changes in histone acetylation, promoter occupancy, and the recruitment of transcription factors and co-regulators to the *LHB* and *FSHB* promoters [82]. Furthermore, longer-acting regulatory inputs, such as follistatin, activin, and inhibin, influence FSH production by modifying FSHβ transcription without the need for acute GnRH stimulation. The slower oscillations in circulating FSH, as opposed to the quick, pulse-synchronous bursts of LH, are also caused by variations in mRNA stability and protein half-life [83].

These temporal differences allow LH and FSH to exert coordinated yet functionally distinct roles. The recruitment and selection of follicles in females is controlled by dynamic FSH modulation, but luteal development, ovulatory cascade activation, and steroidogenesis all depend on LH for timing signals [84]. Although gonadotropin secretion is relatively steady in males, the processes of FSH-mediated Sertoli cell support and LH-driven intratesticular testosterone generation continue to be interdependent. This temporal coordination may be disrupted by altered GnRH pulse patterns, impaired intracellular signal decoding, or inappropriate feedback signaling, leading to defective spermatogenesis, abnormal folliculogenesis, or anovulation.

**Figure 3:**
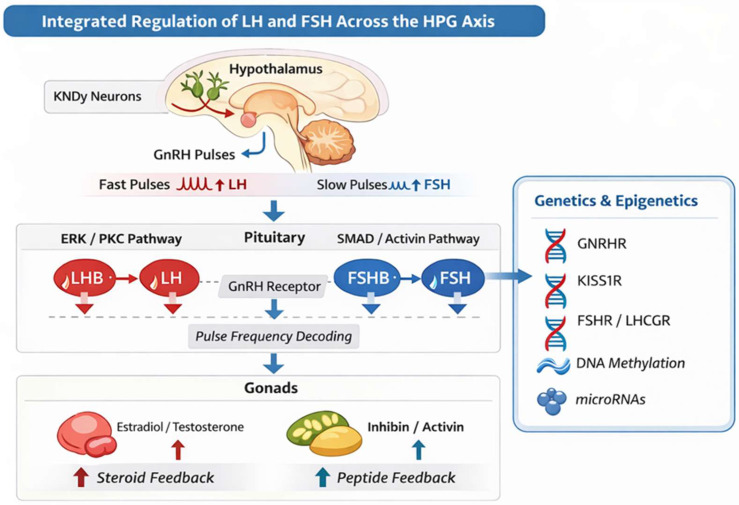
Integrated regulation of LH and FSH across the HPG axis. GnRH pulsatility acts as a frequency-encoded signal regulating pituitary gonadotroph function, with fast pulses promoting LH secretion (via ERK/PKC pathways) and slower pulses promoting FSH production (via SMAD signaling). Gonadal steroids and inhibin/activin provide feedback at hypothalamic and pituitary levels. Genetic and epigenetic modifiers influence multiple levels of this regulatory network, contributing to variability in gonadotropin secretion and reproductive phenotypes. Abbreviations: LH, luteinizing hormone; FSH, follicle-stimulating hormone; HPG, hypothalamic–pituitary–gonadal; GnRH, gonadotropin-releasing hormone; ERK, extracellular signal-regulated kinase; PKC, protein kinase C; SMAD, small mothers against decapentaplegic (SMAD family proteins).

The significance of temporal control in gonadotropin biology is further highlighted by genetic variation that affects transcriptional regulation and pulse decoding components. Gonadotroph responsiveness to pulsatile stimulation can be altered by variations in GNRHR that change receptor desensitization kinetics, polymorphisms affecting the signaling efficacy of the ERK pathway, and mutations in transcription factors like EGR1 or regulatory regions within the *LHB* and *FSHB* promoters. Furthermore, interindividual differences in LH/FSH secretion patterns and susceptibility to reproductive diseases may be influenced by epigenetic regulators that affect chromatin accessibility at gonadotropin gene loci [85]. Therefore, preserving reproductive homeostasis requires accurate molecular interpretation of pulsatile GnRH input [5,86]. An integrated overview of the regulatory mechanisms governing LH and FSH secretion across the HPG axis is presented in Figure 3, highlighting the interplay between GnRH pulsatility, pituitary signal decoding, and gonadal feedback, as well as the influence of genetic and epigenetic modifiers. These mechanisms provide the basis for understanding how dysregulation arises within the HPG axis.

## 4. Mechanisms Leading to Dysregulated LH and FSH Secretion

Disruption at any level of the HPG axis can impair GnRH, pituitary, or gonadal signaling, thereby disturbing LH and FSH homeostasis. Complex endocrine abnormalities often arise from interactions among these disorders [87]. Epigenetic regulation may also contribute to persistent alterations in gonadotropin secretion by modifying transcriptional responses at multiple levels of the HPG axis.

Importantly, these alterations rarely occur in isolation but instead reflect the integration of multiple dysregulated mechanisms across the hypothalamic, pituitary, and gonadal levels. While classical models have described these defects in a linear manner, emerging evidence supports a more interconnected framework in which GnRH pulsatility, intracellular signaling pathways, and feedback mechanisms dynamically interact. However, much of the current mechanistic understanding is derived from experimental or animal-based studies, and its direct translation to human physiology remains limited [1].

Furthermore, several aspects of LH/FSH dysregulation remain incompletely understood or debated, including the relative contribution of altered GnRH pulse generation versus pituitary signal decoding, and the extent to which epigenetic changes represent primary pathogenic mechanisms or secondary adaptive responses. These uncertainties underscore the need for integrative human studies combining endocrine, genetic, and molecular data to better define the mechanisms underlying reproductive dysfunction [88].

### 4.1. Altered GnRH Pulse Generation

The disturbance of GnRH pulsatility is the most prevalent upstream mechanism. Changes in GnRH dynamics directly affect the balance of LH/FSH because pulse frequency differentially affects the transcription of *FSHB* and *LHB*. Accelerated GnRH pulse frequency, characteristic of polycystic ovarian syndrome (PCOS), impairs follicular maturation by preferentially enhancing ERK-dependent LHB transcription while suppressing FSH synthesis [6]. Conversely, loss of GnRH pulsatility in congenital GnRH deficiency or FHA suppresses gonadotropin production, leading to gonadal quiescence and reduced GNRHR signaling. These disorders are mechanistically caused by dysregulation of KNDy neuron activity, modified kisspeptin signaling, and metabolic stress-induced reduction in KISS1 expression [5,89].

In addition to functional and metabolic abnormalities, inherited mutations in genes regulating GnRH neuron development and signaling can impair pulsatility. Patients with congenital hypogonadotropic hypogonadism and delayed puberty have been found to have pathogenic variations in *KISS1*, KISS1R, *TAC3*, TACR3, *GNRH1*, and GNRHR, indicating that disruption of genetically encoded pulse-generation pathways directly affects LH and FSH secretion. These results highlight the possibility that basic genetic abnormalities, rather than only acquired neuroendocrine dysregulation, may be the cause of aberrant GnRH dynamics [12,90].

### 4.2. Pituitary Signaling Defects

Defects in downstream transcriptional regulators such as EGR1 or SMAD proteins, GNRHR mutations, or compromised Gq/11–PLC–PKC signaling can all lead to pituitary dysfunction. Modified expression of *LHB*, *FSHB*, or the *CGA* can impair selective gonadotropin production. Desensitization and reduced LH and FSH secretion result from prolonged non-pulsatile GnRH stimulation, which also induces receptor internalization, β-arrestin recruitment, and receptor phosphorylation. Reduced gonadotropin responsiveness in some clinical circumstances may be caused by epigenetic changes that impact the promoters of gonadotropin genes [46,91,92,93].

This process is further supported by monogenic abnormalities that impact pituitary signaling components. While inactivating mutations in *LHB*, *FSHB*, or *CGA* cause isolated gonadotropin deficits marked by decreased or physiologically inactive hormone production, loss-of-function mutations in GNRHR affect receptor trafficking or intracellular signaling. The hereditary component of pituitary-level dysfunction may also be strengthened by variations in transcriptional regulators or SMAD pathway genes that impair selective gonadotropin production [35].

### 4.3. Feedback and Intragonadal Dysregulation

Disruption of peptide and steroid feedback loops can also result in gonadotropin imbalance. The hypothalamic and pituitary ERα- and PR-mediated feedback is altered by decreased progesterone or estradiol synthesis, which destabilizes the structure of GnRH pulses and the emergence of LH surges. Men who produce less testosterone have fewer androgen receptors to provide negative feedback, which may lead to compensatory increases in LH or inadequate stimulation in central disorders [43,94].

Particularly, modifications to the activin-inhibin-follistatin system affect FSH regulation. Sertoli or granulosa cell failure is characterized by reduced inhibin B secretion, which increases SMAD2/3 signaling and may result in excessive FSH output. Conversely, weakened activin signaling may limit *FSHB* transcription even in the presence of adequate GnRH stimulation [95,96,97].

Dysregulated gonadotropin production is also a result of genetic changes that impact feedback regulators. While variations in activin receptor genes (*ACVR2A*, *ACVR2B*) or SMAD signaling components can change FSH regulation, mutations in *ESR1*, *ESR2*, or *AR* affect steroid receptor–mediated transcriptional feedback. Furthermore, gonadal responsiveness may be compromised by pathogenic variations in FSHR or LHCGR, resulting in compensatory changes in LH and FSH secretion that represent defective feedback integration [98,99].

### 4.4. Downstream Consequences

Insufficient gonadal LH signaling impairs spermatogenesis by reducing intratesticular testosterone levels and the production of steroidogenic enzymes. Women’s variations in LH/FSH ratios impact FSHR and LHCGR signaling in developing follicles, interfering with aromatase activity, ovulatory cascade activation, and luteal function. Therefore, abnormalities in GnRH pulse generation, receptor signaling integrity, or feedback control that propagate through intracellular pathways impair gametogenesis and fertility [100,101].

These downstream effects are often caused by genetic defects in steroidogenic enzymes and gonadotropin receptors. Inactivating mutations in *FSHR* are associated with ovarian insufficiency or reduced spermatogenic efficiency, whereas *LHCGR* mutations may result in Leydig cell hypoplasia or impaired ovulatory signaling. Variants that impact enzymes like *CYP19A1*, *CYP17A1*, or *CYP11A1* may change steroid biosynthesis, intensifying the effects of upstream signaling abnormalities on reproduction. These genotype-phenotype correlations show how clinically different reproductive problems result from molecular abnormalities within the HPG axis [102,103].

The HPG axis’ susceptibility to molecular and neuroendocrine disruptions is highlighted by these processes taken together. To restore physiological gonadotropin dynamics, appropriate therapeutic approaches must be identified, which requires an understanding of how particular signaling disorders affect LH/FSH balance [104].

**Figure 4:**
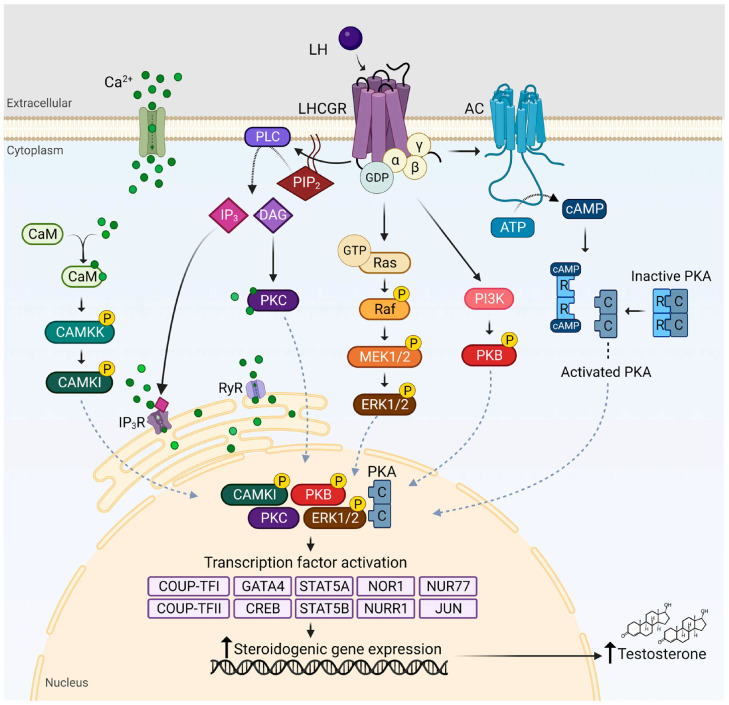
LH signaling pathways in Leydig cells. LH binding to LHCGR activates G protein–mediated signaling pathways, including cAMP/PKA, PLC/PKC, and MAPK cascades. These pathways regulate transcription factors and promote steroidogenic gene expression, leading to testosterone production [71]. Abbreviations: LH, luteinizing hormone; LHCGR, luteinizing hormone/choriogonadotropin receptor; cAMP, cyclic adenosine monophosphate; PKA, protein kinase A; PLC, phospholipase C; PKC, protein kinase C.

Importantly, genotype–phenotype correlations within the HPG axis exhibit significant sex-specific heterogeneity, reflecting fundamental differences in gonadal physiology and feedback regulation. For example, inactivating variants in FSHR are strongly associated with primary ovarian insufficiency and impaired folliculogenesis in females, whereas in males they often result in variable spermatogenic defects with partially preserved fertility [105]. Similarly, mutations in LHCGR may lead to ovulatory dysfunction and luteal insufficiency in females, but cause Leydig cell hypoplasia and severe androgen deficiency in males. Variants affecting central regulators such as *GNRHR* or *KISS1R* may also present with differing clinical severity between sexes, influenced by differential sensitivity of hypothalamic–pituitary feedback loops and gonadal steroid environments. These observations underscore that the clinical expression of genetic defects in gonadotropin regulation is not uniform, but is modulated by sex-specific endocrine context. At the gonadal level, LH binding to LHCGR on Leydig cells activates multiple intracellular signaling pathways, including cAMP/PKA, PLC/PKC, and MAPK cascades [71]. These pathways regulate steroidogenic enzyme activity and gene expression, promoting testosterone production and supporting spermatogenesis (Figure 4). Building on these physiological mechanisms, several pathways contribute to dysregulated LH and FSH secretion.

## 5. Clinical Consequences

The disruption of the closely coordinated molecular pathways controlling gametogenesis and steroidogenesis is reflected in the clinical signs of dysregulated LH and FSH secretion. Given the dependence of gonadotropin production on feedback regulation, receptor signaling, and GnRH pulse decoding, disruption of the HPG axis can lead to diverse reproductive phenotypes [100,104].

Anovulation is a typical manifestation of disrupted LH/FSH balance in women. Defective dominant follicle selection, reduced granulosa cell aromatase activity, and relative FSH insufficiency are the outcomes of PCOS, which is characterized by an accelerated GnRH pulse frequency and preferred *LHB* transcription [106,107]. However, decreased activation of *GNRHR* signaling, decreased expression of *LHB* and *FSHB*, ovarian quiescence, and hypoestrogenism result from suppression of GnRH pulsatility in functional hypothalamic amenorrhea. Luteinization can be compromised even during ovulatory cycles by insufficient LH surge amplitude or compromised *LHCGR* signaling, which can lead to luteal phase insufficiency, subfertility, or early pregnancy loss. Endometrial disease and extended unopposed estrogen exposure may also be predisposed by chronic gonadotropin imbalance [108,109].

These female reproductive phenotypes are largely influenced by hereditary factors in addition to functional endocrine disorders. While mutations in *LHCGR* may affect ovulatory signaling and luteal function, variations in FSHR have been linked to ovarian resistance and primary ovarian insufficiency. Rare mutations in *LHB* or *FSHB* have been connected to isolated gonadotropin shortages that manifest as anovulation or delayed puberty, while pathogenic changes in *CYP19A1* can decrease aromatase activity and estrogen production [110].

The main symptom of dysregulated FSH and LH secretion in males is poor spermatogenesis. While inadequate FSH signaling jeopardizes Sertoli cell support and inhibin B synthesis, reduced LH signaling lowers intratesticular testosterone by restricting StAR and steroidogenic enzyme activity. Depending on their severity, these alterations may show up as azoospermia, asthenozoospermia, or oligozoospermia. In central hypogonadotropic circumstances, low gonadotropin production results in reduced testicular volume and impaired secondary sexual characteristics [111,112].

Male gonadotropin-related infertility is increasingly known to have genetic origins. While pathogenic variations in LHCGR reduce Leydig cell responsiveness, mutations in these genes can cause solitary gonadotropin insufficiency or congenital hypogonadotropic hypogonadism. Mutations in *AR* result in variable degrees of androgen insensitivity and impaired feedback regulation, whereas pathogenic variants in *FSHR* may compromise Sertoli cell signaling and spermatogenic efficiency [12,113].

Long-term hypoestrogenism or hypogonadism affects fertility, but it also has systemic effects like lower bone mineral density, metabolic problems, and changed cardiovascular risk profiles. Thus, the clinical manifestations of LH and FSH dysregulation, which extend beyond the gonads, reflect the broader endocrine roles of sex hormones [114]. Collectively, these findings underscore the clinical relevance of physiological gonadotropin dynamics and highlight the contribution of molecular disruptions in GnRH signaling and feedback regulation to reproductive failure.

## 6. Therapeutic Approaches

Restoring natural GnRH pulsatility, normalizing gonadotropin receptor signaling, or compensating for impaired gonadal feedback are the objectives of treatment strategies that target dysregulated LH and FSH secretion. The following approaches include both established clinical therapies and emerging strategies that remain largely experimental. Since dysfunction may start at the hypothalamus, pituitary, or gonadal level, therapeutic approaches should be customized to the particular genetic abnormality within the HPG axis [115]. When GnRH pulsatility is impaired, the simplest course of action is to restore physiological stimulus. Pulsatile GnRH stimulation of pituitary gonadotrophs restores GnRH receptor signaling, enabling appropriate transcription of *LHB* and *FSHB* and coordinated secretion of LH and FSH. Clinically, this approach is effective in treating congenital GnRH deficiency and functional hypothalamic amenorrhea, restoring ovulation in women and spermatogenesis in men when pituitary responsiveness is preserved [116,117]. Genetic characterization may guide treatment decisions in individuals with known mutations in *GNRHR*, *KISS1R*, or other genes affecting central GnRH pulse generation by predicting pituitary responsiveness to pulsatile GnRH therapy. Gonadotropin secretion recovery may vary in people with partial loss-of-function mutations or receptor trafficking abnormalities, highlighting the importance of genotype-informed treatment approaches.

When direct gonadotropin control is required or the pituitary response is inadequate, exogenous gonadotropin therapy is employed. Ovarian follicles’ *FSHR* and *LHCGR* signaling is stimulated by recombinant FSH or combination FSH/LH preparations, which increases aromatase activity, follicular maturation, and ovulation. By activating LHCGR in Leydig cells, hCG increases the production of testosterone in boys with hypogonadotropic hypogonadism. Then, spermatogenic development and Sertoli cell activity are enhanced by recombinant FSH [70,73,118]. Individual reactivity to exogenous gonadotropins may be influenced by genetic variation in *FSHR* and *LHCGR*. Pharmacogenetic profiling may optimize dosage regimens and enhance reproductive outcomes because some receptor variants have been linked to varied ovarian stimulation outcomes, variable estradiol production, and variations in spermatogenic response.

Upstream metabolic and neuroendocrine regulators are the main focus of treatment strategies for conditions like polycystic ovarian syndrome, which are associated with excessive LH pulsatility [7]. GnRH pulse frequency and LH secretion can be indirectly normalized by improving insulin sensitivity. By lowering estrogen-mediated negative feedback, ovulation stimulation with aromatase inhibitors, including letrozole, increases endogenous FSH secretion [119].

As long as reproduction is not the primary goal and gonadal steroid production is inadequate, hormone replacement therapy is still necessary. To maintain bone, metabolic, and cardiovascular health, systemic endocrine balance is restored by estrogen–progesterone therapy for women and testosterone replacement for men [120].

**Figure 5:**
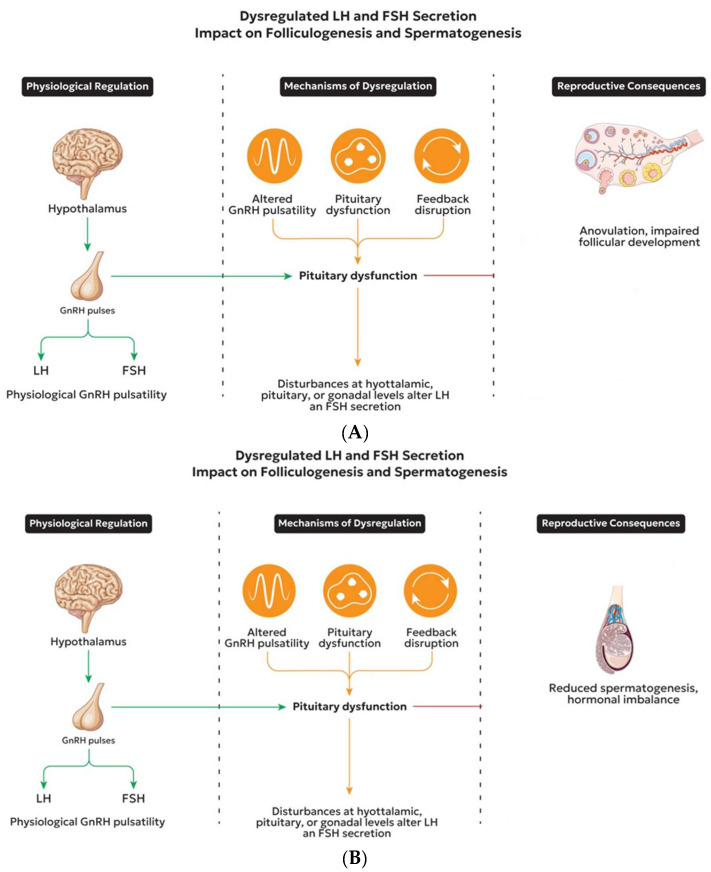
(**A**). Dysregulation of LH and FSH secretion and its impact on female reproductive function. Physiological GnRH pulsatility regulates LH and FSH secretion from the pituitary. Disruption of this system may occur at multiple levels: (1) altered GnRH pulsatility, leading to preferential LH secretion and relative FSH deficiency; (2) pituitary dysfunction, impairing gonadotropin synthesis and secretion; and (3) disrupted gonadal feedback involving steroid hormones and the activin–inhibin system. These alterations result in impaired follicular development, anovulation, and reproductive dysfunction. (**B**). Dysregulation of LH and FSH secretion and its impact on male reproductive function. Physiological GnRH pulsatility regulates LH and FSH secretion from the pituitary. Disruption may occur at multiple levels: (1) altered GnRH pulsatility; (2) pituitary dysfunction affecting gonadotropin synthesis and secretion; and (3) impaired gonadal feedback mechanisms mediated by androgens and intragonadal signaling pathways. These disturbances lead to reduced spermatogenesis, impaired testosterone production, and hormonal imbalance. Abbreviations: GnRH, gonadotropin-releasing hormone; LH, luteinizing hormone; FSH, follicle-stimulating hormone.

Advances in mechanistic understanding have led to the development of targeted therapies, including modulation of the activin–inhibin–SMAD axis to regulate FSHB expression and the use of kisspeptin analogs to restore physiological GnRH neuron activity [121,122]. Future precision therapies that restore gonadotropin dynamics at their biological source may be made possible by ongoing developments in our understanding of intracellular signal decoding and receptor modulation. An improved understanding of the molecular architecture of the reproductive axis has driven a shift toward targeted modulation of specific signaling pathways in the management of gonadotropin imbalance [123]. Emerging gene-targeted and epigenetic treatments represent potential future developments in reproductive endocrinology. Advances in transcriptomic profiling, gene editing, and genomic sequencing may enable correction of pathogenic variants or modulation of dysregulated gene expression within the HPG axis. These approaches may, in the future, complement or refine empirical hormone replacement strategies as genotype–phenotype associations become more thoroughly characterized. However, these strategies remain at an early stage and require further validation before clinical application. These pathophysiological mechanisms (Figure 5A,B) provide the basis for targeted therapeutic strategies.

## 7. Future Perspectives and Emerging Directions

Progress in molecular endocrinology has reshaped current concepts of LH and FSH regulation and dysregulation. Functional heterogeneity among pituitary gonadotroph populations, including differential expression patterns of *LHB*, *FSHB*, *GNRHR*, and related transcriptional regulators, is starting to be revealed by emerging single-cell transcriptomic and epigenomic techniques [124,125]. These technologies may elucidate how pathological states alter promoter accessibility and transcription factor recruitment, as well as how pulse frequency is decoded at the chromatin level. Whole-exome and whole-genome sequencing are increasingly uncovering rare and common variants implicated in reproductive endocrine disorders. Integrating genomic and single-cell epigenomic data may reveal how inherited variants modulate chromatin architecture and pulse decoding in gonadotroph subsets.

One particularly intriguing field of study is epigenetic regulation. Long-term regulation of *KISS1*, *GNRH1*, and gonadotropin subunit genes has been associated with DNA methylation, histone modifications, and non-coding RNAs, particularly in the context of metabolic stress, chronic inflammation, or androgen excess. Clarifying these processes may help explain why reproductive dysfunction persists even after systemic endocrine balance is restored [17,126,127]. Moreover, interactions between genes and the environment are becoming important factors in determining the results of reproduction. The clinical manifestation of conditions like PCOS or hypogonadotropic hypogonadism may be exacerbated or lessened by epigenetic changes interacting with underlying genetic susceptibility factors. Longitudinal epigenomic research could be useful in differentiating between heritable regulatory changes and reversible adaptive modifications.

Additionally, mechanism-based precision techniques are becoming more prevalent in therapeutic development. Without constant receptor activation, kisspeptin analogs and neurokinin receptor modulators may be able to restore normal GnRH pulsatility [128]. Simultaneously, more precise regulation of *FSHB* transcription and FSH secretion may be possible through targeting the activin–inhibin–SMAD signaling axis [59,129,130]. To further enhance customized reproductive therapy, genetic screening for variations in *GNRHR*, *FSHR*, or *LHCGR* may be integrated. Incorporating comprehensive genomic panels into reproductive endocrinology practice may allow early detection of harmful mutations and support genotype-guided therapeutic techniques as sequencing technology become more widely available. In the longer term, targeted correction of specific molecular flaws within the HPG axis may become possible due to advancements in gene-editing platforms and RNA-based therapies; however, safety and ethical concerns will always be paramount [131]. Preclinical studies further support the feasibility of these approaches. Viral vector–mediated gene delivery has been shown to restore components of GnRH signaling pathways in animal models of hypogonadotropic hypogonadism [132], while RNA interference (RNAi) strategies have been explored to modulate the expression of key regulators involved in gonadotropin synthesis and steroidogenesis [133,134]. Although these interventions remain experimental, they highlight the potential for directly correcting underlying molecular defects within the HPG axis.

Together, these new approaches show how the HPG axis is shifting from empirical hormonal replacement to targeted intracellular signaling and gene regulatory control [98,131].

## 8. Conclusions

The ability of humans to reproduce depends on the precise coordination of LH and FSH secretion. Gonadotropin balance is maintained via transcriptional regulation of *LHB* and *FSHB*, intracellular kinase signaling integration, differential decoding of GnRH pulsatility, and carefully controlled steroid and peptide feedback mechanisms. Anovulation, luteal insufficiency, poor spermatogenesis, and hypogonadism are among the distinguishing reproductive abnormalities that result from disruption of these molecular networks, whether at the level of gonadal feedback, pituitary signal transduction, or GnRH pulse production.

Pathogenic polymorphisms that impact GnRH signaling, gonadotropin subunits, receptor function, and steroid feedback pathways directly contribute to a variety of reproductive abnormalities in addition to functional dysregulation, according to mounting genetic data. These genotype–phenotype connections highlight the possibility that inherited molecular abnormalities within the HPG axis may cause disturbance of LH and FSH balance, underscoring the necessity of an integrated genetic and endocrine assessment.

This review uniquely integrates intracellular signal decoding mechanisms with genetic determinants of HPG axis dysfunction, providing a mechanistic framework that links molecular alterations to distinct reproductive phenotypes and therapeutic variability. By combining molecular, cellular, and clinical findings, it illustrates how abnormalities in specific signaling pathways propagate throughout the reproductive axis to impair fertility. Understanding SMAD-mediated transcriptional regulation, receptor kinetics, pulse frequency decoding, and steroid receptor cross-talk in greater detail offers a molecular basis for diagnosing endocrine diseases and creating tailored treatments. Instead of merely substituting hormones, future therapeutic strategies will increasingly rely on molecular precision to restore physiological signaling architecture within the HPG axis.

Despite these advances, important gaps remain, including limited direct characterization of GnRH pulsatility in humans, incomplete understanding of the relative contribution of central versus pituitary mechanisms, and insufficient integration of genetic and epigenetic modifiers in clinically heterogeneous conditions. Addressing these challenges through integrative, systems-level approaches will be essential for translating mechanistic insights into personalized therapeutic strategies in reproductive endocrinology.

## Data Availability

No new data were created or analyzed in this study.
